# Needle-free delivery of measles virus vaccine to the lower respiratory tract of non-human primates elicits optimal immunity and protection

**DOI:** 10.1038/s41541-017-0022-8

**Published:** 2017-08-01

**Authors:** Rik L. de Swart, Rory D. de Vries, Linda J. Rennick, Geert van Amerongen, Stephen McQuaid, R. Joyce Verburgh, Selma Yüksel, Alwin de Jong, Ken Lemon, D. Tien Nguyen, Martin Ludlow, Albert D. M. E. Osterhaus, W. Paul Duprex

**Affiliations:** 1 Department of Viroscience, Erasmus MC, Rotterdam, The Netherlands; 2 Department of Microbiology, Boston University School of Medicine, Boston, MA USA; 3 Queen’s University of Belfast, Belfast, Northern Ireland UK; 4 Viroclinics Biosciences, Rotterdam, Netherlands; 5 Present Address: ProQR Therapeutics, Leiden, Netherlands; 6 Present Address: Agri-Food and Biosciences Institute, Belfast, UK; 7 Present Address: University of Veterinary Medicine Hannover, Hannover, Germany

## Abstract

Needle-free measles virus vaccination by aerosol inhalation has many potential benefits. The current standard route of vaccination is subcutaneous injection, whereas measles virus is an airborne pathogen. However, the target cells that support replication of live-attenuated measles virus vaccines in the respiratory tract are largely unknown. The aims of this study were to assess the in vivo tropism of live-attenuated measles virus and determine whether respiratory measles virus vaccination should target the upper or lower respiratory tract. Four groups of twelve cynomolgus macaques were immunized with 10^4^ TCID_50_ of recombinant measles virus vaccine strain Edmonston-Zagreb expressing enhanced green fluorescent protein. The vaccine virus was grown in MRC-5 cells and formulated with identical stabilizers and excipients as used in the commercial MV^EZ^ vaccine produced by the Serum Institute of India. Animals were immunized by hypodermic injection, intra-tracheal inoculation, intra-nasal instillation, or aerosol inhalation. In each group six animals were euthanized at early time points post-vaccination, whereas the other six were followed for 14 months to assess immunogenicity and protection from challenge infection with wild-type measles virus. At early time-points, enhanced green fluorescent protein-positive measles virus-infected cells were detected locally in the muscle, nasal tissues, lungs, and draining lymph nodes. Systemic vaccine virus replication and viremia were virtually absent. Infected macrophages, dendritic cells and tissue-resident lymphocytes predominated. Exclusive delivery of vaccine virus to the lower respiratory tract resulted in highest immunogenicity and protection. This study sheds light on the tropism of a live-attenuated measles virus vaccine and identifies the alveolar spaces as the optimal site for respiratory delivery of measles virus vaccine.

## Measles: The efficacy of needle-free vaccine delivery

Research shows that measles aerosol vaccination is effective when the vaccine is delivered to the lower respiratory tract. Measles virus is a highly contagious and potentially deadly human pathogen, but is easily prevented by vaccination. An international team led by Paul Duprex, of the United States’ Boston University School of Medicine, and Rik de Swart, of the Erasmus University Medical Center, The Netherlands, investigated the effectiveness of different routes of measles vaccine administration in non-human primates. They found that macaques responded well to aerosolized administration of the live-attenuated measles vaccine and they were effectively protected from subsequent measles virus infection. Vaccine delivery to the lower respiratory tract was most effective in eliciting protective immune responses. This research is a step towards easier vaccination protocols and away from dependency on sterile needles and trained health-care workers — an important consideration in the developing world.

## Introduction

Measles remains a leading cause of childhood morbidity and mortality. Live-attenuated measles virus (MV) vaccines are safe and effective, and high coverage in two-dose regimens has successfully interrupted endemic transmission in large geographic areas.^1^ However, measles still caused an estimated 134,200 deaths in 2015,^2^ and vaccine refusal based on religious and/or philosophical objections has led to measles resurgence in Europe and the Americas.^3, 4^

Wild-type MV is one of the most contagious human pathogens, is spread by airborne transmission and causes an acute systemic disease.^5^ Measles is associated with transient but severe immune suppression, which results in increased susceptibility to opportunistic infections.^6, 7^ Live-attenuated MV vaccines were introduced in the early 1960s at a time when worldwide millions of children were dying of measles every year. Introduction of MV vaccination has consistently reduced childhood mortality in every geographic region.^8^ Despite their success, live-attenuated MV vaccines also have limitations, including dependency on maintaining the cold-chain, requirement for trained health-care workers and the need for sterile hypodermic needles and concomitant safe waste disposal. Needle-free MV vaccination regimens were developed to address some of these issues and administration of the vaccine by aerosol inhalation (AI) has been considered as a promising technology.^9^ Initially, clinical trials using aerosolized measles vaccine were performed in Mexico and these demonstrated the feasibility of this vaccination route.^10^ Recent studies confirmed that aerosol measles vaccination was equivalent or superior to injection as booster vaccine.^11^ However, when used for primary immunization of infants, aerosol vaccination resulted in lower seroconversion levels than standard injection.^12^ A randomized, controlled trial of aerosol vaccination in infants 9–12 months of age confirmed that aerosol vaccination was immunogenic, but aerosol delivery induced lower seroconversion rates than subcutaneous (SC) injection (85% and 95%, respectively).^13^

Regulatory authorities consider a vaccine and its administration route as a single entity. Consequently, both preclinical and clinical studies were required to achieve licensure for MV aerosol vaccination. To support licensing we previously compared administration of the MV Edmonston-Zagreb (MV^EZ^) vaccine strain via AI, dry powder inhalation and injection in non-human primates.^14^ Aerosol vaccination induced similar levels of neutralizing antibodies and T-cell responses as detected in the injection group, and protection from challenge infection with a wild-type MV was comparable. Dry powder aerosol vaccination was less effective than injection in this study.^15^ However, in a more recent study successful immunization of macaques by an aerosolized dry powder MV vaccine was reported,^16^ and this approach was shown to be safe in a phase I clinical trial in humans.^17^

Despite the fact that live-attenuated MV vaccines have been used for almost 50 years, there is still a fundamental lack of understanding of the in vivo tropism of the virus and the molecular basis of attenuation remains elusive. This hampers rational development of next-generation delivery strategies that could be transformative in the developing world by mitigating challenges associated with virus thermostability. Live-attenuated MV must replicate in the host to induce protective immune responses, and alternative routes of administration may well deliver the vaccine to different target cells than those infected following injection. In recent years, we have used recombinant (r) MV strains expressing fluorescent proteins to study wild-type MV tropism in vitro and in vivo.^18–22^ These viruses express a fluorescent reporter protein from an additional transcription unit (ATU), which has the advantage that the entire cytoplasm of an infected cell is flooded with the reporter proteins facilitating sensitive detection of infected cells by flow cytometry, immunofluorescence and/or immunohistochemistry.^23^ We recently described an rMV based on the MV^EZ^ vaccine strain expressing enhanced green fluorescent protein (EGFP), which was produced using a miniaturized (laboratory-scale) industrial process using cells and protocols provided by the Serum Institute of India. This reporter virus permitted detection of small numbers of MV-infected cells in the muscle of vaccinated non-human primates in the first week following injection.^24^ In addition, we optimized methodology for dose estimation and aerosol delivery in non-human primates.^25^ Based on these foundational studies we now report results of a large-scale vaccination study in cynomolgus macaques comparing four different routes of immunization. The aim of the study was to compare vaccine virus replication, tropism, immunogenicity and protection, and determine whether MV vaccination via the respiratory route should target the upper or lower respiratory tract (LRT).

## Results

### Study design

**Fig. 1:**
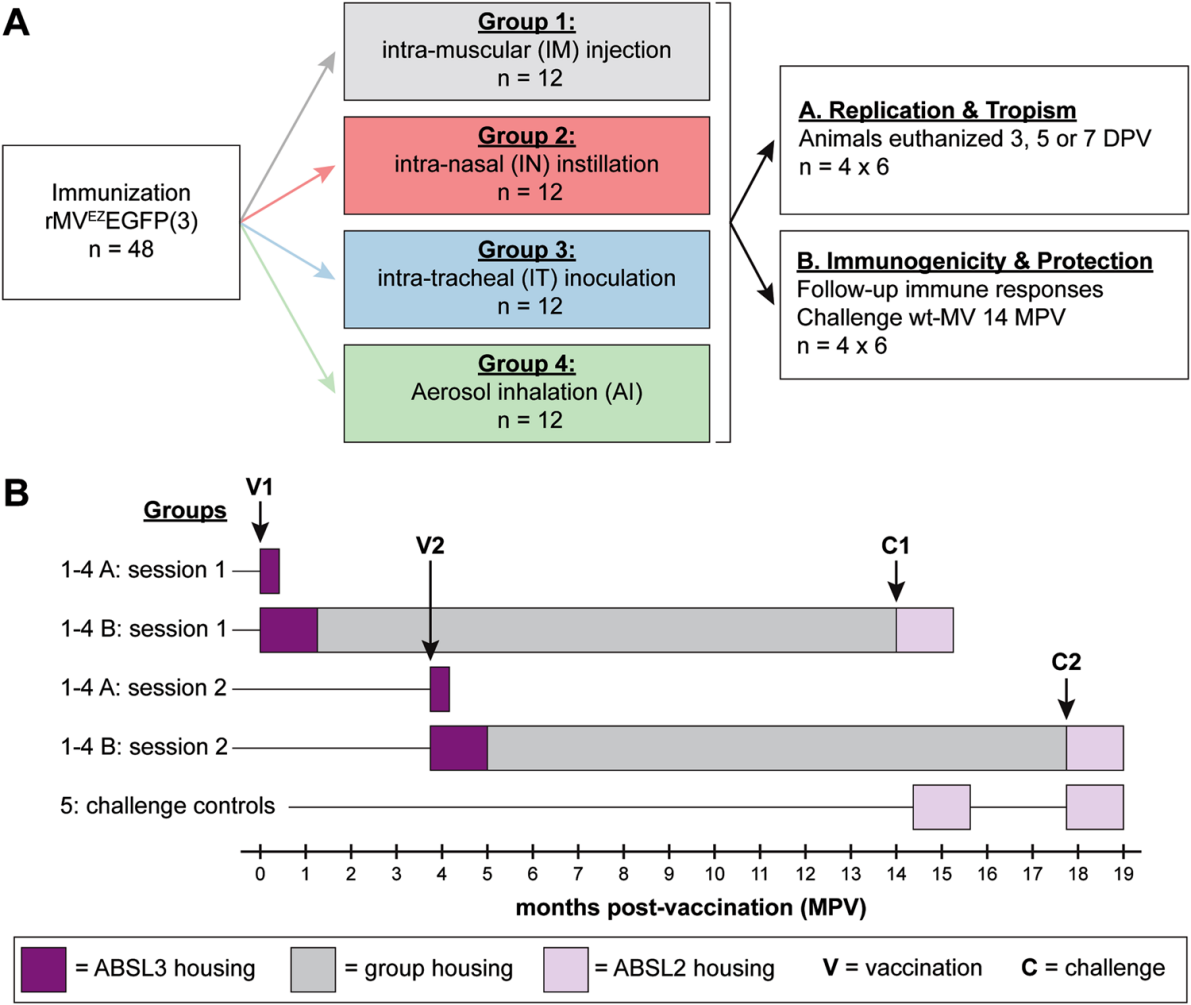
Study design. **a** Schematic representation of the experimental groups included in the vaccination study. All animals received the same dose of the same vaccine, administered via four different routes. Colors used for the four treatment groups correspond to symbol colors (Figs. 2, 3, 4, and 5). A detailed list of animals and samples is provided in Table S1. **b** For logistical reasons, the experiment was performed in two parallel sessions (1 and 2), as illustrated. Each session included six animals of each group, of which three were euthanized at 3, 5, or 7 DPV for assessment of replication and tropism (subgroups **a**), and three were followed up for assessment of immunogenicity and protection (subgroups **b**). The time points of vaccination (V) and challenge (C) are indicated by V1/V2 and C1/C2, respectively. *DPV* days post-vaccination, *MPV* months post-vaccination. Two groups of two seronegative animals were included as challenge controls at C1 and C2

Four groups of 12 cynomolgus macaques were immunized by intra-muscular (IM) injection, intra-tracheal (IT) inoculation, intra-nasal (IN) instillation, or AI of rMV^EZ^EGFP(3).^24^ Six animals from each group were euthanized at early time points in the virus replication and tropism arm of the study, the other six were followed-up for 14 months post-vaccination (MPV) to assess immunogenicity and protection from wild-type MV challenge infection (Fig. 1a). For logistical reasons the study was performed in two parallel sessions (1 and 2) comprising half of the animals from each of the groups (Fig. 1b and Supplementary Table 1). To comply with Dutch laws on the use of genetically modified organisms, vaccinations were performed in high efficiency particulate air (HEPA)-filtered negatively pressurized animal biosafety level (ABSL)-3 isolators.

### Replication

**Fig. 2:**
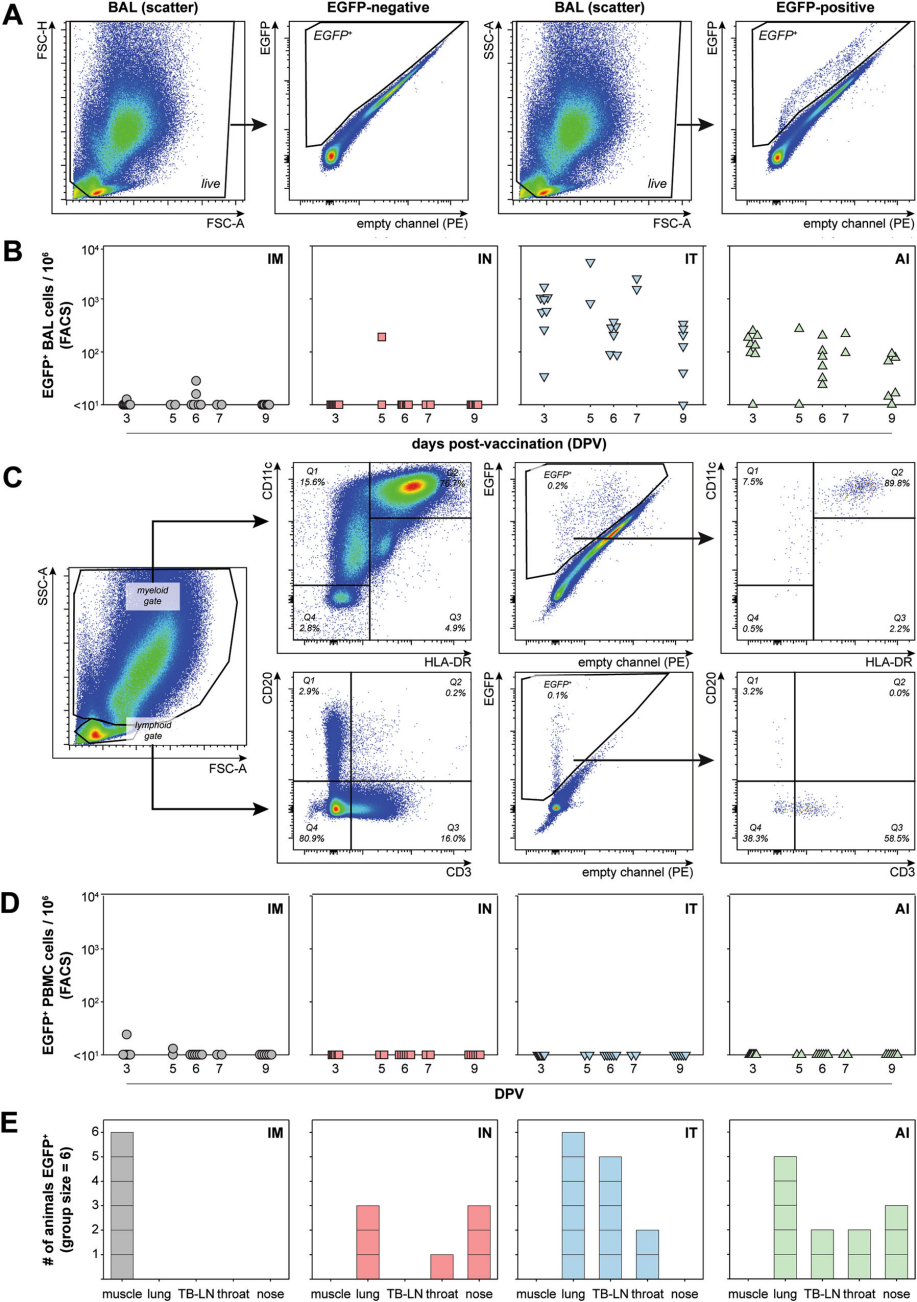
Replication. Detection of EGFP-positive cells in clinical specimens collected from non-human primates immunized with rMV^EZ^EGFP(3) via four different routes of administration. **a** Detection of EGFP-positive cells in BAL cells by flow cytometry: examples of a negative (*left*) and positive (*right*) sample. Please note that the *narrow diagonal band* represents non-specific autofluorescent cells, the EGFP-positive events are contained in the triangular gate in the *upper left* of the PE—EGFP plots. **b** Based on these FACS analyses, frequencies of EGFP-positive BAL cells are shown for animals in the four treatment groups (including animals of both subgroups **a** and **b**). **c** FACS staining of a representative EGFP-positive BAL cell sample. Two cell populations were distinguishable in scatter plots: cells with large FSC and SSC values (myeloid gate) predominantly contained CD11c^+^ and HLA-DR^+^ myeloid cells while cells with low FSC and SSC values (lymphoid gate) contained CD20^+^ B-lymphocytes and CD3^+^ T-lymphocytes but in majority (approximately 80%) were not phenotyped. Reverse gating of the EGFP-positive myeloid and lymphoid cells showed that these predominantly were CD11c^+^HLA-DR^+^ myeloid and CD3^+^ lymphoid cells, respectively. **d** Extremely low to undetectable levels of EGFP-positive cells in PBMC indicated absence of viremia after vaccination. **e** Detection of EGFP-positive cells in muscle (by UV microscopy, as reported previously),^24^ lung (fluorescence microscopic screening of lung slices as described previously),^58^ TB-LN (> 10 EGFP-positive cells per 10^6^ TB-LN, similar to panels **b** and **d**), throat (virus isolation), or nose (combination of virus isolation and UV microscopy screening of tissues collected (Fig. S1). Each block represents one positive animal. *BAL* broncho-alveolar lavage, *PBMC* peripheral blood mononuclear cells, *TB-LN* tracheo-bronchial lymph node

Clinical specimens collected during the first 9 days post-vaccination (DPV) were screened for the presence of MV-infected (EGFP-positive) cells by combining flow cytometry (fluorescence-activated cell sorting (FACS)), ultraviolet (UV) microscopy and virus isolation to assess vaccine virus replication. FACS analysis of unstained broncho-alveolar lavage (BAL) cells was a sensitive method for detection of EGFP-positive cells in the LRT, as illustrated by a negative and positive sample at five DPV (Fig. 2a). The highest frequencies of EGFP-positive BAL cells were consistently detected in animals of groups 3 (IT) and 4 (AI), while infected cells were undetectable in the BAL of most animals in groups 1 (IM) or 2 (IN) (Fig. 2b). This was compatible with virus isolation data obtained from BAL cells: in 24 out of 48 animals EGFP-positive BAL cells were detected by FACS analysis, and we were able to isolate MV from BAL cells from 23 out of these 24 animals, predominantly at 3, 5, or 6 DPV (results not shown).

FACS scatter plots of BAL samples showed that at least two separate populations of cells were present in BAL, one with low forward scatter (FSC) and side scatter (SSC), compatible with lymphocyte scatter in FACS analysis of peripheral blood mononuclear cell (PBMC) and lymph node samples, and the second with higher FSC and SSC values (Fig. 2c, *left* panel). A number of BAL samples containing EGFP-positive cells were stained with cell-specific antibodies to assess the phenotype of the MV-infected cells. The lymphoid fraction contained B-cells (CD20^+^), T-cells (CD3^+^) as well as CD3^−^CD20^−^ cells, but the majority of the EGFP-positive BAL cells in the lymphoid fraction were T-lymphocytes (Fig. 2c). The BAL cell population with high FSC/SSC mostly contained MV-infected myeloid CD11c^+^HLA-DR^+^ cells, most likely alveolar macrophages or dendritic cells (Fig. 2c).

A similar FACS analysis to that used for BAL cells was performed using unstained PBMC, collected at different DPV from animals in both arms of the study. With the exception of two samples collected from animals in group 1 (IM), frequencies of EGFP-positive events in PBMC were consistently below 10 per million events (Fig. 2d). These observations are consistent with virus isolation data: all attempts to isolate vaccine virus from PBMC at multiple DPV were unsuccessful.

Muscle slices, prepared from animals euthanized 3, 5, or 7 DPV, were screened by UV microscopy to detect replicating rMV^EZ^EGFP at the site of administration. As reported previously, we detected EGFP-positive cells interdigitating the musculature at the injection site of all six animals in group 1 (IM), and in none of the animals vaccinated via the respiratory routes (Fig. 2e).^24^ Lung slices were prepared from animals euthanized 3, 5, or 7 DPV to assess MV infection of the LRT. These were screened for EGFP-positive cells by UV microscopy and high frequencies of MV-infected cells were detected in 6/6 and 5/6 animals of groups 3 (IT) and 4 (AI), respectively (Fig. 2e). In group 2 (IN), the lungs of one animal contained several foci of MV-infected cells (compatible with detection of EGFP-positive BAL cells in the same animal, see Fig. 2b), while in the lungs of two other animals in this group only a single EGFP-positive cell was detected. Infected cells were not detected in the lungs of other animals in groups 1 and 2. Nasal tissues (nasal septum, nasal concha, and nasal epithelium) were analyzed systematically by UV microscopy to detect MV-infected cells in the upper respiratory tract (URT, see explanation of sample collection in Fig. S1). In addition, virus isolation was performed from throat and nasal swabs. MV-infected cells were only detected in the nose and throat of animals immunized via respiratory routes (groups 2–4), although not in all animals of these groups (Fig. 2e). Absolute numbers of EGFP-positive cells detected in the URT of animals immunized by IN instillation or AI were substantially lower than those detected in the LRT of animals immunized via the IT or AI routes. In conclusion, MV vaccination by injection or delivery to the LRT resulted in detection of EGFP-positive cells in samples consistent with the route of delivery.

### Tropism

Targeted pathological assessment of a wide range of formalin-fixed samples collected during necropsy was used for further assessment of viral tropism. This approach was straightforward at the peak of wild-type MV infection, but proved more challenging upon MV vaccination due to the limited numbers of EGFP-positive cells. UV microscopy-positive samples were dissected, formalin-fixed and paraffin-embedded (FFPE) and multiple 7 µm sections were prepared from leveled tissues to permit small numbers of EGFP-positive cells to be identified.

**Fig. 3:**
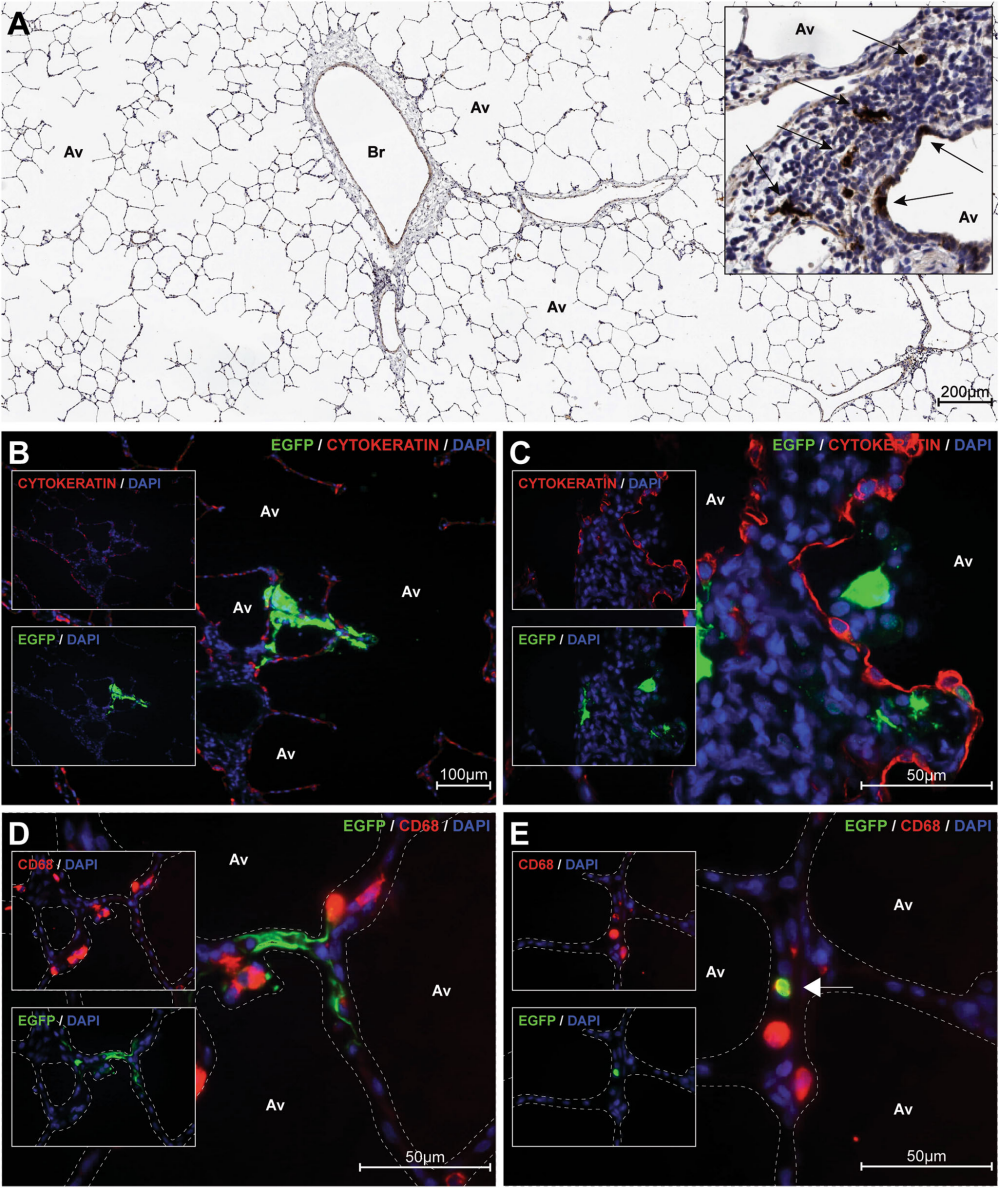
Tropism. Macaques were euthanized by exsanguination 3, 5, or 7 DPV. During necropsy, lungs were inflated with low-melting point agarose and allowed to solidify on ice. Lung slices were screened by UV microscopy for the presence of EGFP-positive cells. Seven micrometer sections were prepared from leveled, fixed, and paraffin embedded tissues to permit small numbers of EGFP-positive cells to be identified. **a** Hematoxylin counterstained anti-EGFP immunohistochemical staining of a lung slice, showing evidence of EGFP-positive cells in *brown* (see *inset*, positive cells indicated by arrows); **b**, **c** EGFP (*green*)/4’,6-diamidino-2-phenylindole (*DAPI*; *blue*) / cytokeratin (*red*) staining (single fluorescence scans shown in *insets*); **d**, **e**: EGFP (*green*)/DAPI (*blue*)/CD68 (*red*) staining (single fluorescence scans shown in *insets*). *Arrow* in panel **e** indicates CD68 and EGFP co-localization. Images shown were collected from animal T-41 (see Table S1, group 4 A (AI), euthanized 5 DPV). *Av* alveoli, *Br* bronchiole

Since formalin fixation destroys EGFP autofluorescence, serial sections were prepared and one in every ten was stained with an anti-EGFP monoclonal antibody and HRP-conjugated secondary antibody and counterstained with hematoxylin (Fig. 3a). In most FFPE slices we were unable to detect MV-infected cells, but in a few samples MV-infected cells were identified in the lung sections. Serial sections of these slices were used for dual immunofluorescent labeling. MV-infected cells were mainly cytokeratin-negative (Fig. 3b, c), and some MV-infected cells expressed CD68 demonstrating a macrophage lineage (Fig. 3d, e). Equivalent MV-infected cells were present in nasal epithelia in animals from group 2 (IN; data not shown). In conclusion, MV vaccine virus predominantly replicated in myeloid and lymphoid cells and rarely in epithelial cells.

### Immunogenicity

**Fig. 4:**
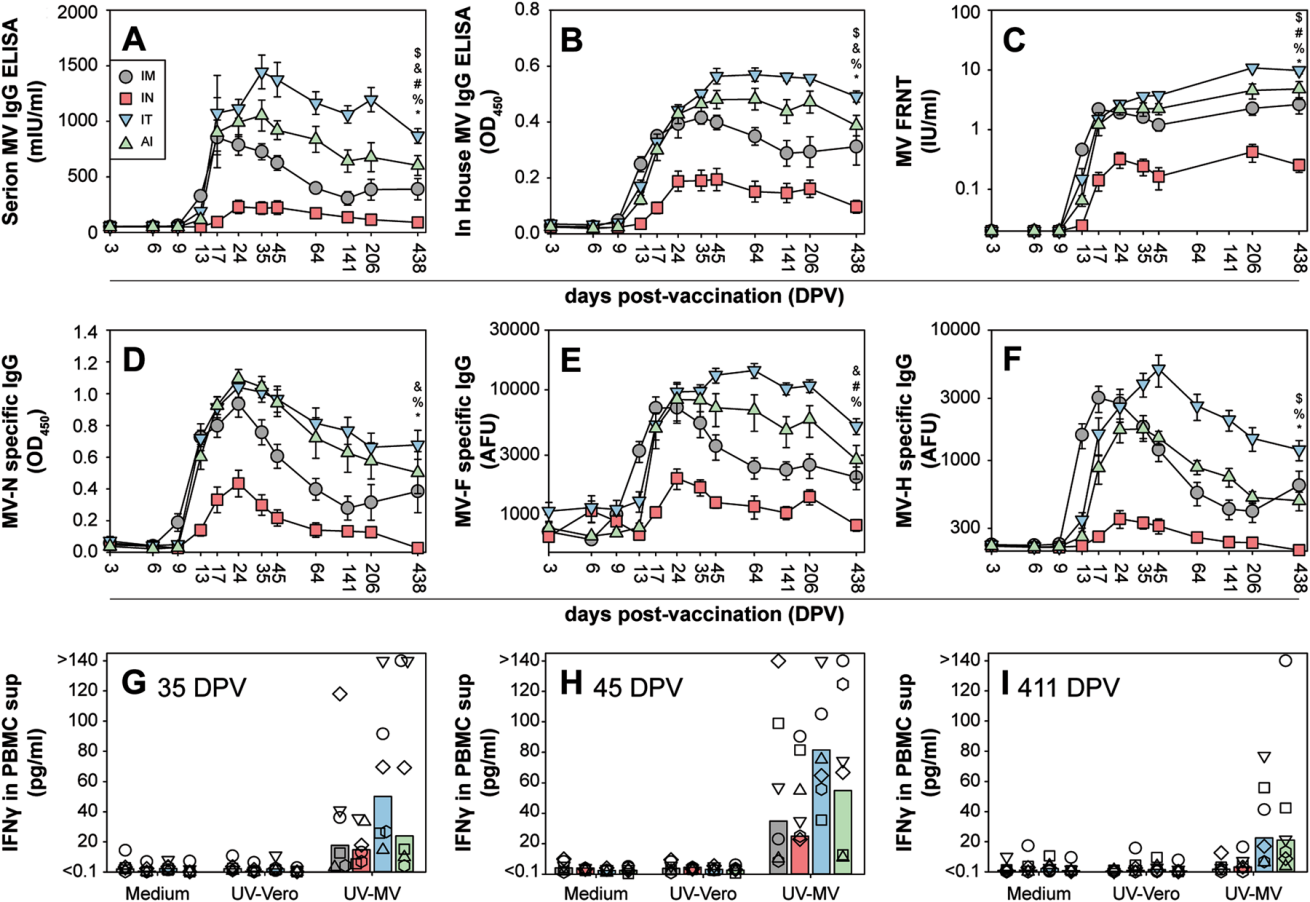
Immunogenicity. Virus-specific immune responses after immunization of non-human primates with rMV^EZ^EGFP(3) via four different routes of administration. Panels **a**–**f** show MV-specific serum antibody responses measured by six different assays: **a** Serion IgG ELISA, with results expressed in milli-international units per ml (mIU/ml); **b** in house MV IgG ELISA, with results expressed in optical density at 450 nm (OD_450_); **c** focus reduction neutralization test (*FRNT*), with results expressed in international units per ml (IU/ml); **d** MV nucleoprotein (*MV*-*N*)-specific IgG ELISA, with results expressed in OD_450_; **e** MV fusion (MV-F) glycoprotein-specific FACS-measured IgG immunofluorescence (expressed in arbitrary fluorescence units, AFU); **f** MV hemagglutinin (*MV*-*H*) glycoprotein-specific FACS-measured IgG immunofluorescence (expressed in AFU). Results are shown as means ± standard error of six animals per group. Panels **g–i** show MV-specific cellular responses measured using PBMC collected 35 (**g)**, 45 (**h**), or 411 (**i**) DPV. Briefly, PBMC were stimulated with UV-inactivated MV (UV-MV), mock antigen (UV-Vero) or medium. Culture supernatants were harvested after 2 days for measurement of the concentration of IFN-γ by ELISA. *Bars* represent the means, while *symbols* represent responses of individual animals. Results of statistical analysis (Two-way analysis of variance with Tukey’s multiple comparison test) are indicated by the following symbols if *P*-values were below 0.05: $ (IM vs. IT),! (IM vs. AI), and (IM vs. IN), # (IT vs. AI), % (IT vs. IN), * (AI vs. IN)

Animals were followed for MV-specific immune responses for 14 MPV. Serum antibody responses were detected by enzyme linked immunosorbent assay (ELISA, Fig. 4a, b, d), virus neutralization (Fig. 4c) or indirect immunofluorescence for MV fusion (MV-F) or hemagglutinin (MV-H) glycoprotein-specific antibodies (Fig. 4e, f). Animals vaccinated by IT inoculation (blue symbols) consistently showed highest antibody levels, whereas samples from AI administered (green symbols) and IM (grey symbols) injected animals showed slightly lower responses. In all assays, lowest serum antibody levels were consistently observed in animals immunized by IN instillation (red symbols). Serological profiles were comparable to cellular immune responses measured at 35, 45, or 411 DPV: in vitro PBMC stimulation assays consistently resulted in highest levels of interferon-gamma (IFN-γ) production in supernatants of cells collected from animals in groups 3 (IT) and 4 (AI), and lowest responses in animals of group 2 (IN, Fig. 4g–i). In conclusion, vaccine delivery to the LRT resulted in higher immunogenicity than vaccination by IM injection, but exclusive delivery to the URT consistently resulted in lower immune responses.

### Protection

**Fig. 5:**
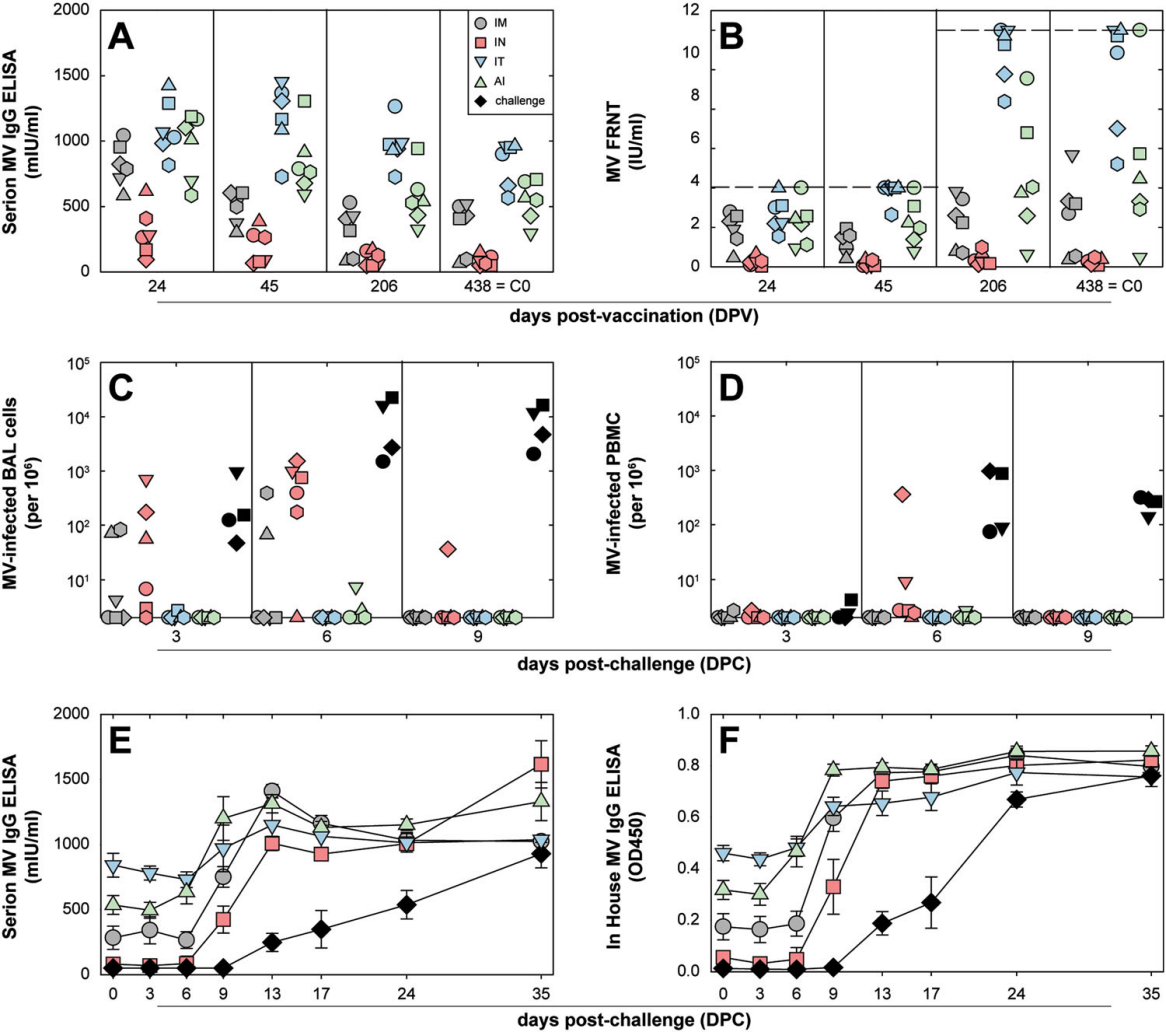
Protection. Immunization of non-human primates with rMV^EZ^EGFP(3) via four different routes of administration induced varying levels of protection to IT challenge with wild-type MV strain Bilthoven 14 MPV. In panels **a**–**d** symbols represent individual animals: **a**, **b** show MV-specific serum antibody levels before challenge as measured by ELISA or FRNT, respectively (in panel **b** the *dotted lines* show the upper limit of detection of the two assays that were performed); **c**, **d** show frequencies of wild-type MV-infected BAL cells and PBMC after challenge infection, respectively. Panels **e**, **f** show MV-specific serum antibody levels after challenge, demonstrating accelerated secondary immune responses in all vaccinated animals as compared to slow primary responses in the challenge control animals (means ± standard error per group). Symbol colors and shapes for vaccinated animals are identical to those in Figs. 1–4, *black* symbols represent challenge controls

Fourteen MPV, all twenty-four animals in the immunogenicity arm of the study, and four additional seronegative control animals, were challenged with wild-type MV strain Bilthoven by IT inoculation with a dose of 10^4^ TCID_50_ (based on titration in Vero-hCD150 cells)^26^ as described previously.^27^ At the moment of challenge infection (438 DPV = 0 days post-challenge; DPC), MV-specific serum antibody levels in the majority of vaccinated animals were above previously identified immunological correlates of protection of 120−200 mIU/ml^28^ (Fig. 5a, b).

Frequencies of MV-infected BAL cells and PBMC were measured using an infectious center assay to assess the level of protection to challenge infection (Fig. 5c, d). As expected the highest virus loads were detected in challenge control animals, although high levels were also detected in animals immunized by IN instillation (group 2), and two out of six animals immunized by IM injection (which also had the lowest serum antibody levels at time point C0) also showed substantial virus loads in BAL cells collected 3 or 6 DPC. Only two out of six IN immunized animals had detectable virus loads in PBMC. Measurement of MV-specific serum antibody responses at different time points post-challenge (Fig. 5e, f) demonstrated accelerated secondary immune responses in all vaccinated animals as compared to slower primary responses in the challenge control animals (black symbols). Animals in group 4 (AI) showed the most rapid onset of a secondary immune response, with four out of six animals showing strong increases in specific antibody levels between 3 and 6 DPC (Fig. S2). In conclusion, all vaccinated animals were largely protected from systemic wild-type MV replication, but also showed secondary immune responses demonstrating the absence of sterilizing immunity after MV vaccination.

One animal (I–31; group 3, session 1, IT) failed to recover from anesthesia after collection of clinical specimens 9 DPC. A full necropsy was performed to establish a possible cause of death. Replicating MV was not detected and no gross pulmonary lesions were observed. Histopathological evaluation showed a very mild neutrophilic tracheitis and bronchitis that may have been associated with the MV-specific immune response. No lesions in any tissues were found that could explain the cause of death. This led to the conclusion that the unexpected death resulted from anesthesia.

In conclusion, immunogenicity and protection, as determined by inhibition of wild-type MV replication, were closely related. Lowest relative protection levels (as shown by challenge virus replication) were detected in animals immunized by IN instillation and in two out of six animals immunized by IM injection. Delivery of MV vaccine to the LRT (groups 3 and 4) resulted in high immunogenicity and this was associated with high levels of protection from wild-type MV replication.

## Discussion

Live-attenuated virus vaccines have had a profound impact on human and animal health. Development of measles vaccines started 50 years ago, based on the first MV isolate that was adapted to grow on embryonated chicken eggs and embryo fibroblasts.^29^ Measles vaccines are safe and effective and have successfully interrupted endemic MV transmission in large parts of the world.^30^ However, neither the molecular basis of attenuation nor the in vivo tropism of live-attenuated measles vaccine viruses are completely understood.

We have previously used rMVs generated using wild-type MV sequences obtained directly from clinical specimens, and therefore unmodified by tissue culture adaptation, for in vitro and in vivo pathogenesis studies. An ATU encoding a fluorescent reporter protein had a negligible effect on virulence and allowed sensitive detection of infected cells. Studies employing these rMVs have improved our understanding of wild-type MV entry,^21, 31^ dissemination^19, 20^ and transmission.^32^ Furthermore, these viruses were essential in elucidating how wild-type MV causes immune suppression.^7^ The possibility of identifying single MV-infected cells as early as 2 days post-infection in tissues and organs^21^ suggested that it is possible to perform tropism studies following vaccination. This approach was piloted following immunization of macaques by IM injection and permitted the identification of the primary target cells in the muscle.^24^ Here we extended this approach, and identified the tropism of measles vaccine viruses after IN instillation, IT inoculation or AI. To facilitate these studies we optimized aerosol administration and dose estimation of MV vaccine to non-human primates.^25^

Repeated tissue culture passage of wild-type MV, which normally uses CD150 or nectin-4 as cellular entry receptors,^33, 34^ resulted in attenuated MV vaccine strains that may use the ubiquitously expressed protein CD46 as an entry receptor.^35, 36^ Even though CD46 is used by live-attenuated MV in vitro, careful pathological assessments show scant evidence that this is the case in vivo.^20, 24, 37^ Here, we show that rMV^EZ^ was predominantly detected in CD3^+^ T-lymphocytes, major histocompatibility complex (MHC) class II^+^CD11c^+^ dendritic cells and CD68^+^ macrophages after delivery of live-attenuated virus to the respiratory tract. These cell types all express CD150 showing infection by vaccine viruses is mediated by the primary entry receptor for wild-type MV. Evidence for in vivo usage of CD46, i.e., infection of CD150^-^/nectin-4^-^ cells, was not obtained during these studies even though there are enormous numbers of CD46-positive cells in close proximity to MV-infected centers. Collectively, these data demonstrate the importance of using relevant animal models of vaccination to determine the in vivo tropism of live attenuated vaccine viruses, rather than relying on extrapolations from in vitro data.

Since wild-type MV is transmitted via the respiratory route, it has been suggested that immunization via the airways might improve vaccine immunogenicity and efficacy compared to injection.^38^ Initial clinical trials using aerosolized measles vaccine were performed in Mexico and these demonstrated the feasibility of this route of delivery.^10^ Subsequent pre-clinical and clinical studies confirmed vaccination efficacy and demonstrated no evidence for serious adverse events.^9^ Moreover, aerosol measles vaccination might result in improved mucosal immune responses,^39^ similar to other respiratory pathogen vaccines.^40–42^

The potential of delivering measles vaccine as an aerosol, alongside the desire to reduce and/or eliminate needle use in the developing world, led us to perform a unique study in non-human primates comparing the immunogenicity of rMV^EZ^ delivered via different routes of respiratory administration. The particle size of the aerosol (mass median aerodynamic diameter 3.5 µm) was chosen to allow deposition of a substantial fraction of the inhaled dose into the LRT.^25^ Robust humoral immune responses were induced by IM injection, IT inoculation and AI, whereas IN instillation induced poor antibody responses. Furthermore, direct delivery of live-attenuated vaccine to the LRT via IT inoculation or AI gave superior humoral and cellular responses compared to IM injection. These results demonstrate that the immunogenicity of MV^EZ^ after primary immunization with equivalent dosage was comparable when the vaccine was delivered by AI or injection.^14^

All animals vaccinated via the IT and AI route still had MV-specific, neutralizing antibodies at the moment of challenge infection at 14 MPV. Interestingly, these antibodies had waned in 6/6 IN vaccinated animals and 2/6 IM vaccinated animals. This was reflected post-challenge and infectious virus was more likely to be recovered from BAL cells obtained from animals without detectable antibodies (6/6 IN, 2/6 IM, 0/6 IT, 0/6 AI and 4/4 challenge controls). A significant boosting of antibodies, characteristic of a secondary immune response, was observed in all vaccinated animals and this was both faster and stronger than the primary immune responses observed in the challenge control animals. Rapid increases in MV-specific antibodies was most evident in animals vaccinated via AI. Although this study was not designed to assess vaccine safety, it is important to note that no indications for adverse events associated with these non-conventional vaccination routes were noted.

Even though aerosol delivery of live-attenuated MV vaccine offers promise,^38, 43, 44^ clinical studies in children have produced variable results. Although effective in inducing secondary immune responses as booster vaccination,^45^ primary vaccination of infants proved inferior to vaccination by standard injection,^13, 46, 47^ contradictory to the results we obtained in non-human primates. Extending exposure time of infants to aerosol resulted in improved responses,^48^ suggesting delivery of insufficient vaccine as a potential explanation for the reduced effectiveness in infants with low tidal breathing volumes and irregular breathing patterns. These issues, and the fact that in our study immunogenicity of IT inoculation was superior to AI, highlight the need to determine the parameters for normalization of the dose delivered to the LRT during aerosol delivery, if the goal of aerosol MV vaccination is ever to be realized.

Dry powder inhalation has also been examined as an alternative to nebulized AI.^16, 17, 49, 50^ This approach avoids the need for vaccine reconstitution and allows packaging of individual doses in a thermostable formulation, which may reduce dependence on cold chain maintenance. Microneedle patches containing measles vaccine are considered as another alternative, and have similar advantages.^51^ Patches contain a stabilized formulation of vaccine that dissolves in the skin minutes after application, thereby avoiding the use of hypodermic needles. Microneedle delivery patches were immunogenic in macaques,^52^ and studies with a combination of microneedle patches and an rMV vaccine strain encoding a fluorescent reporter protein could support further development and elucidate the tropism of live-attenuated MV vaccine in the dermis and/or epidermis of the skin.

Since live-attenuated virus vaccines typically contain a low antigenic dose, replication in the host is essential for induction of protective immune responses. Primary vaccine virus replication in professional antigen presenting cells (expressing MHC class II) in the LRT, as we show here, could facilitate the induction of robust immune responses. This demonstrates that it is critical to understand replication and tropism of existing live-attenuated vaccines, as this will inform the optimal route of delivery of vaccines. In conclusion, we show that delivery of live-attenuated MV vaccines to the LRT is a biologically feasible method of measles vaccination. These studies support the notion that inferior immunogenicity following primary immunization of infants can be attributed to suboptimal vaccine virus delivery to the LRT in the short exposure time used in this trial.^13^ It remains to be seen whether the collective will exists to re-evaluate respiratory MV vaccination in clinical studies in infants.

## Methods

### Ethics statement

Animal experiments were conducted in compliance with European guidelines (EU directive on animal testing 86/609/EEC) and Dutch legislation (Experiments on Animals Act, 1997). The study protocol was approved by Stichting Dier Experimenten Commissie Consult (DEC Consult, permit number EMC2646), a Dutch independent animal experimentation ethics review board. Animals were housed in groups, received standard primate feed and fresh fruit on a daily basis and had access to water ad libitum. In addition, their cages contained several sources of environmental enrichment, for example hiding places, hanging ropes, tires, and other toys. During the vaccination and challenge periods (0–24 DPV and 0–24 DPC) animals were housed in HEPA-filtered, negatively pressurized ABSL-3 isolator cages, both to ensure biological containment of the genetically modified viruses and to reduce stress during the repeated sample collection by using the squeeze mechanism for sedating the animals. Animal welfare was observed on a daily basis, and all animal handling was performed under light anesthesia using ketamine and medetomidine (50/50 v/v, 0.2 ml/kg body weight, IM injection). After handling, atipamezole was administered to antagonize the effect of medetomidine (0.05 ml/kg body weight, IM injection). The manuscript was prepared in accordance with the “Animal Research: Reporting of In Vivo Experiments” (ARRIVE) guidelines.

### Animal study design

Forty-eight male cynomolgus macaques (*Macaca fascicularis*) were immunized with 10^4^ TCID_50_ of rMV^EZ^EGFP(3) administered via four different routes of administration: IM injection (*n* = 12), IN instillation (*n* = 12), IT inoculation (*n* = 12) or AI (*n* = 12) (Fig. 1a).

The standard route of MV vaccination is SC injection. However, the skin of macaques is more loosely connected to the subcutus than the skin of humans, therefore, we chose to use IM rather than SC injection to avoid redistribution of the vaccine over a large surface area. Several studies have demonstrated that both routes are safe and effective in humans.^53, 54^ IM injection was performed in 0.5 ml into the *rectus femoris*. IN instillation was included as a route of administration to assess tropism and immunogenicity if the complete vaccine dose was delivered to the URT only. IN instillation (0.5 ml) was performed by using a Gilson P200 pipette, dividing the volume over both nostrils. Immediately after instillation, the nostrils were gently squeezed from the outside to distribute the vaccine virus over the surfaces of the nasal cavity. IT instillation was included as a route of administration to assess tropism and immunogenicity if the complete vaccine dose was delivered to the LRT. Immediately before IT instillation the vaccine virus was diluted 1:10 in phosphate-buffered saline (PBS, Gibco), and 5 ml was inoculated IT through a flexible catheter inserted into the trachea just below the larynx. AI was performed as described.^25^ Briefly, vaccine virus was nebulized using a vibrating mesh nebulizer (Aeroneb Pro, Aerogen Ireland Ltd., mass median aerodynamic diameter 3.5 µm), connected to a 22 mm T-piece and using a silicone pediatric resuscitation mask (Laerdal 0/0) as interface with the macaque. Bench studies performed using macaque breathing parameters demonstrated that approximately 25% of a nebulized dose was inhaled by the animal, whereas the remainder of the dose was lost as condensate in the nebulizer or T-piece or was exhaled. Therefore, 0.5 ml of a 4× concentrated vaccine virus stock was nebulized to deliver the same vaccine dose as in the other three groups. It should be noted that there is no control over the fate of the inhaled vaccine virus after inhalation: part may be lost when it gets swallowed, whereas the remainder will be deposited in the URT or the LRT.

Six animals of each of the four vaccination groups were used to study viral tropism. These were euthanized 3 (*n* = 2), 5 (*n* = 2), or 7 (*n* = 2) DPV. The other six animals/group were followed up for 14 months to study immunogenicity, and were subsequently challenged with wild-type MV. An additional four unvaccinated animals were included as challenge controls. Due to the limitations of housing facilities and laboratory capacity, the experiment was performed in two parallel sessions with separate vaccination and challenge days but sticking to identical time intervals. The study design is summarized in Fig. 1, animal numbers, characteristics, vaccination routes, and sample collection time points are listed in Supplementary Table 1.

### Sample size estimation, randomization, and blinding

In the replication and tropism arm of the study, the main outcome parameters were qualitative assessment and characterization of EGFP-positive cells at early time points after vaccination. We selected three time points of euthanasia (3, 5 and, 7 DPV) based on the expected peak of virus replication as determined in previous measles vaccination studies in the macaque model, and chose to include two animals at each time point (leading to a total group size of 6). In the immunogenicity and protection arm of the study, the required group size was based on a power calculation using MV-specific serum antibody responses as main outcome parameter. Based on variation in antibody responses in previous non-human primate vaccination studies, we estimated that a group size of six animals would result in adequate statistical power.

Upon arrival from the supplier, animals were randomly assigned to groups of three animals. These groups were maintained as much as possible for the complete duration of the study, and only modified in the beginning if animals were not socially compatible. Stable groups of non-human primates should not be changed, and, therefore, the same group composition was used when the animals were moved into DM-III isolators for their vaccinations. Which isolator was used for each route of administration was selected randomly (without using a formal randomization procedure).

During sample collection, tubes were labeled with the last four digits of the animal identification chip number, and subsequently transported to the lab. Laboratory technicians were not aware which animal number corresponded to which of the different treatment groups.

### Viruses

Animals were vaccinated with rMV^EZ^EGFP(3). Non-recombinant vaccine virus, MRC-5 cells and protocols for vaccine production and formulation were kindly provided by the Serum Institute of India. Generation, formulation, and characterization of the recombinant vaccine virus was described previously.^24^ The formulated vaccine virus had a history of one passage in Vero-hCD150 cells (rescue) and five passages in MRC-5 cells, and was stored at −80 °C. The stock had a titer of 10^6.6^ TCID_50_/ml, and was diluted in formulated medium^24^ to 10^4^ TCID_50_ per 0.5 ml. Challenge infections were performed by IT inoculation with wild-type (non-recombinant) MV strain Bilthoven, as described previously.^14, 27^

### Samples

Small-volume EDTA blood samples were collected in Vacuette tubes containing K_3_EDTA as an anticoagulant. Plasma was separated from the blood by centrifugation, heat inactivated (30 min; 56 °C), and stored at −20 °C. PBMC were isolated from EDTA blood by density gradient centrifugation and resuspended in complete RPMI 1640 medium (Gibco Invitrogen, Carlsbad, CA) supplemented with l-glutamine (2 mM), 10% (v/v) FBS, penicillin (100 U/ml), and streptomycin (100 μg/ml). BAL was performed in animals of the immunogenicity arm at 3, 6, and 9 DPV and 3, 6, and 9 days DPC by IT infusion of 10 ml PBS through a flexible catheter followed by immediate recovery of the fluid. BAL samples were centrifuged, and BAL cells were resuspended in culture medium with supplements as described above. Throat brushes and nasal swabs were collected in virus transport medium as described elsewhere.^19^ For the virus tropism study, animals were euthanized by exsanguination under deep anesthesia using ketamine and medetomidine. During necropsy, tissue samples were collected in PBS and directly processed and screened for the presence of EGFP by UV microscopy. EGFP-positive samples were either transferred to 4% (w/v) paraformaldehyde in PBS (to preserve EGFP autofluorescence) for direct imaging, to 10% (v/v) neutral-buffered formalin for paraffin embedding or to the laboratory for preparation of single-cell suspensions for flow cytometry. Direct imaging was performed by confocal laser scanning microscopy with a LSM700 system fitted on an Axio Observer Z1 inverted microscope (Zeiss), and images were generated using Zen software version 2010B SP1.

### Virus detection

Isolation of MV vaccine or wild-type virus was performed on Vero or Vero-hCD150 cells (kind gift of Dr Y. Yanagi, Fukuoka, Japan),^26^ respectively. All cell lines tested negative for mycoplasma spp. contamination. Virus isolation was performed by infectious-center test as previously described,^55^ monitoring for cytopathic effect and/or fluorescence 6 days post-isolation. Results of vaccine virus isolations are shown as positive or negative (regardless of load), results of wild-type MV isolation are shown as the number of infected cells per million. Flow cytometry was used to detect EGFP-positive MV-infected cells in PBMC or single-cell suspensions of lymphoid tissues. Immunohistochemistry (IHC) was performed as described previously.^24^ Briefly, GFP was visualized in IHC and dual-labeling indirect immunofluorescence with a polyclonal rabbit anti-EGFP (Invitrogen) or rabbit anti-MV (Novus Biologicals) antibody. Monoclonal mouse antibodies to the macrophage marker CD68 (Dako; clone KP1) or the epithelial cell marker Cytokeratin (Abcam; clone Ae1/Ae3) were used to visualize the respective cell types. In dual-labeling immunofluorescence, antigen-binding sites were detected with a mixture of anti-mouse Alexa 568 and anti-rabbit Alexa 488 (Invitrogen). Sections were counterstained with 4’,6-diamidino-2-phenylindole (DAPI) hard-set mounting medium (Vector). Fluorescently stained slides were examined at ×100, ×200, ×400, and ×1000 magnifications on a fluorescence imaging microscope (Leica Microsystems).

### Virus-specific immune responses

MV-specific antibody levels were measured in serum (virus neutralization) or EDTA plasma samples (all other serological assays). The ELISA *classic* Measles Virus IgG assay (Serion, Würzburg, Germany) was performed according to the instructions of the manufacturer. The in house MV-specific IgG ELISA, MV-N-specific IgG ELISA, and the MV-F and MV-H glycoprotein-specific indirect immunofluorescence assays were performed as described previously.^56^ In all cases, bound antibodies were detected by polyclonal anti-human IgG conjugates that show excellent cross-reactivity with macaque IgG.

Virus neutralizing antibodies were detected using a fluorescent focus reduction neutralization assay,^57^ with some modifications. Briefly, Vero-hCD150 cells were seeded in 96-well flat-bottom plates (Greiner) in RPMI-1640 supplemented with 10% (v/v) fetal bovine serum (FBS) 4 days before the experiment, and refreshed with RPMI-1640 medium supplemented with 2% (v/v) FBS (R2F) the day before the experiment. Serial dilutions (2^2^–2^9^, each dilution tested in triplicate) of heat inactivated serum in R2F were mixed 1:1 (v/v) with 40 TCID_50_ of rMV^EZ^EGFP(3), and incubated for 90 min at 37 °C. Subsequently, the serum/virus mixtures were transferred to the Vero-hCD150 monolayers and spinoculated for 15 min at 1200×*g*. Cells were cultured at 37 °C for 2 h, after which fusion inhibitory peptide (FIP: Z-d-Phe-L-Phe-Gly-OH, Bachem, Heidelberg, Germany) was added to a final concentration of 0.2 mM to prevent cell-to-cell fusion. Two days later, plates were washed with PBS, fixed with 1% (w/v) paraformaldehyde in PBS, and single fluorescent cells were counted using an automated fluorescence imager (ImmunoSpot S6 analyzer, CTL, Bonn, Germany). Graphpad Prism 5.01 software was used to calculate sigmoidal dose–response curves, on basis of which a 50% reduction titer (EC50) was determined. Results were expressed in international units per ml using the WHO 3rd international reference serum for measles (NIBSC, South Mimms, United Kingdom).

Purified MV Edmonston with a concentration of approximately 1 mg/ml was inactivated by UV-irradiation (30 min, 15 W 312 nm), for cellular assays and is hereafter referred to as UV-MV. A Vero cell lysate was also UV-irradiated, and used as mock control antigen (hereafter referred to as UV-Vero). PBMC were thawed and allowed to recover overnight at 37 °C in RPMI-1640 supplemented with 10% (v/v) human serum, 1% (v/v) macaque serum, and 3.5 ng/ml recombinant human IL-7 (ImmunoTools, Friesoythe, Germany). The following day cells were counted and plated in 96-well round-bottom plates (Greiner) at a concentration of 3 × 10^5^ cells per well. Triplicate cultures were stimulated with UV-MV (final dilution 1:100), UV-Vero (final dilution 1:100) or medium. Two days later supernatants were harvested and IFN-γ concentrations were measured using a monkey IFN-γ ELISA (U-Cytech, Utrecht, Netherlands).

### Statistical analyses

Longitudinal data were analyzed in GraphPad Prism version 7.0a, using grouped analysis: Two-way analysis of variance for repeated measures with Tukey’s multiple comparisons test. *P*-values lower than 0.05 were considered significant. This generalized linear model was automatically parameterized, resulting in 20 degrees of freedom and *n*-values of 78.

### Data availability

All data generated or analyzed during this study are included in this published article (and its Supplementary Information files).

## Electronic supplementary material

Table S1

Figure S1

Figure S2
