# Supplementary material for: Needle-free delivery of measles virus vaccine to the lower respiratory tract of non-human primates elicits optimal immunity and protection
Source: NPJ Vaccines. 2017 Aug 1;2:22. doi: 10.1038/s41541-017-0022-8 (PMC5627256; doi:10.1038/s41541-017-0022-8)
Supplement: Supplementary file 1 — Table S1 [file 41541_2017_22_MOESM1_ESM.docx]

**Supplementary Table 1: Animals and samples**

Table S1A: replication and tropism

| **Group** | **Route** | **Session** | **Animal** | **BW^1^** | **Age^2^** | **Necropsy** | **Samples (DPV)^3^** |
| --- | --- | --- | --- | --- | --- | --- | --- |
| 1 A | IM^4^ | 1 | T-01 | 5.1 | 68 | 3 DPV | 0, 1, 2, 3 |
| 1 A | IM | 1 | T-02 | 5.4 | 70 | 5 DPV | 0, 1, 2, 3, 4, 5 |
| 1 A | IM | 1 | T-03 | 4.7 | 71 | 7 DPV | 0, 1, 2, 3, 4, 5, 6, 7 |
| 1 A | IM | 2 | T-04 | 3.3 | 36 | 3 DPV | 0, 1, 2, 3 |
| 1 A | IM | 2 | T-05 | 4.5 | 40 | 5 DPV | 0, 1, 2, 3, 4, 5 |
| 1 A | IM | 2 | T-06 | 3.1 | 39 | 7 DPV | 0, 1, 2, 3, 4, 5, 6, 7 |
| 2 A | IN^7^ | 1 | T-13 | 5.1 | 65 | 3 DPV | 0, 1, 2, 3 |
| 2 A | IN | 1 | T-14 | 5.6 | 63 | 5 DPV | 0, 1, 2, 3, 4, 5 |
| 2 A | IN | 1 | T-15 | 4.1 | 73 | 7 DPV | 0, 1, 2, 3, 4, 5, 6, 7 |
| 2 A | IN | 2 | T-16 | 3.3 | 39 | 3 DPV | 0, 1, 2, 3 |
| 2 A | IN | 2 | T-17 | 3.8 | 39 | 5 DPV | 0, 1, 2, 3, 4, 5 |
| 2 A | IN | 2 | T-18 | 3.0 | 34 | 7 DPV | 0, 1, 2, 3, 4, 5, 6, 7 |
| 3 A | IT^5^ | 1 | T-25 | 4.8 | 63 | 3 DPV | 0, 1, 2, 3 |
| 3 A | IT | 1 | T-26 | 4.5 | 65 | 5 DPV | 0, 1, 2, 3, 4, 5 |
| 3 A | IT | 1 | T-27 | 5.5 | 65 | 7 DPV | 0, 1, 2, 3, 4, 5, 6, 7 |
| 3 A | IT | 2 | T-28 | 3.2 | 38 | 3 DPV | 0, 1, 2, 3 |
| 3 A | IT | 2 | T-29 | 3.8 | 40 | 5 DPV | 0, 1, 2, 3, 4, 5 |
| 3 A | IT | 2 | T-30 | 3.3 | 40 | 7 DPV | 0, 1, 2, 3, 4, 5, 6, 7 |
| 4 A | AI^6^ | 1 | T-37 | 5.3 | 68 | 3 DPV | 0, 1, 2, 3 |
| 4 A | AI | 1 | T-38 | 4.8 | 62 | 5 DPV | 0, 1, 2, 3, 4, 5 |
| 4 A | AI | 1 | T-39 | 5.2 | 71 | 7 DPV | 0, 1, 2, 3, 4, 5, 6, 7 |
| 4 A | Ai | 2 | T-40 | 3.7 | 38 | 3 DPV | 0, 1, 2, 3 |
| 4 A | Ai | 2 | T-41 | 3.5 | 40 | 5 DPV | 0, 1, 2, 3, 4, 5 |
| 4 A | Ai | 2 | T-42 | 3.7 | 37 | 7 DPV | 0, 1, 2, 3, 4, 5, 6, 7 |

Table S1B: Immunogenicity and protection

| **Group** | **Route** | **Session** | **Animal** | **BW** | **Age** | **Necropsy** | **Samples (DPV)** | **Samples (DPC)^8^** |
| --- | --- | --- | --- | --- | --- | --- | --- | --- |
| 1 B | IM | 1 | I-07 | 5.3 | 65 | - | -8, 0, 3, 6, 9, 13, 17, 24, 35, 45, 84, 136, 206, 411, 438 | 0, 3, 6, 9, 13, 17, 24, 35 |
| 1 B | IM | 1 | I-08 | 5.2 | 71 | - |  |  |
| 1 B | IM | 1 | I-09 | 5.4 | 71 | - |  |  |
| 1 B | IM | 2 | I-10 | 3.0 | 37 | - | -8, 0, 3, 6, 9, 13, 17, 24, 35, 45, 84, 146, 206, 411, 438 | 0, 3, 6, 9, 13, 17, 24, 35 |
| 1 B | IM | 2 | I-11 | 3.0 | 36 | - |  |  |
| 1 B | IM | 2 | I-12 | 2.8 | 36 | - |  |  |
| 2 B | IN | 1 | I-19 | 5.1 | 76 | - | -8, 0, 3, 6, 9, 13, 17, 24, 35, 45, 84, 136, 206, 411, 438 | 0, 3, 6, 9, 13, 17, 24, 35 |
| 2 B | IN | 1 | I-20 | 5.2 | 74 | - |  |  |
| 2 B | IN | 1 | I-21 | 5.1 | 71 | - |  |  |
| 2 B | IN | 2 | I-22 | 3.2 | 38 | - | -8, 0, 3, 6, 9, 13, 17, 24, 35, 45, 84, 146, 206, 411, 438 | 0, 3, 6, 9, 13, 17, 24, 35 |
| 2 B | IN | 2 | I-23 | 3.0 | 39 | - |  |  |
| 2 B | IN | 2 | I-24 | 3.0 | 36 | - |  |  |
| 3 B | IT | 1 | I-31 | 5.0 | 68 | - | -8, 0, 3, 6, 9, 13, 17, 24, 35, 45, 84, 136, 206, 411, 438 | 0, 3, 6, 9, 13, 17, 24, 35 |
| 3 B | IT | 1 | I-32 | 5.2 | 63 | - |  |  |
| 3 B | IT | 1 | I-33 | 5.4 | 70 | - |  |  |
| 3 B | IT | 2 | I-34 | 3.1 | 40 | - | -8, 0, 3, 6, 9, 13, 17, 24, 35, 45, 84, 146, 206, 411, 438 | 0, 3, 6, 9, 13, 17, 24, 35 |
| 3 B | IT | 2 | I-35 | 3.1 | 39 | - |  |  |
| 3 B | IT | 2 | I-36 | 2.9 | 35 | - |  |  |
| 4 B | AI | 1 | I-43 | 5.3 | 65 | - | -8, 0, 3, 6, 9, 13, 17, 24, 35, 45, 84, 136, 206, 411, 438 | 0, 3, 6, 9, 13, 17, 24, 35 |
| 4 B | AI | 1 | I-44 | 5.1 | 70 | - |  |  |
| 4 B | AI | 1 | I-45 | 5.4 | 70 | - |  |  |
| 4 B | AI | 2 | I-46 | 3.5 | 36 | - | -8, 0, 3, 6, 9, 13, 17, 24, 35, 45, 84, 146, 206, 411, 438 | 0, 3, 6, 9, 13, 17, 24, 35 |
| 4 B | AI | 2 | I-47 | 3.4 | 40 | - |  |  |
| 4 B | AI | 2 | I-48 | 3.2 | 39 | - |  |  |
| 5 B | - | 1 | C-49 | - | 54 | - | - | 0, 3, 6, 9, 13, 17, 24, 35 |
| 5 B | - | 1 | C-50 | - | 63 | - |  |  |
| 5 B | - | 2 | C-51 | - | 39 | - | - | 0, 3, 6, 9, 13, 17, 24, 35 |
| 5 B | - | 2 | C-52 | - | 38 | - |  |  |

*^1^BW = body weight (in kg) at time of vaccination; ^2^age in months at time of vaccination (1-48) or challenge infection (49-52); ^3^DPV = days post-vaccination; ^4^IM = intra-muscular injection; ^5^IT = intra-tracheal inoculation; ^6^AI = aerosol inhalation; ^7^IN = intra-nasal instillation; ^8^DPC = days post-challenge infection: 0 DPC = 438 DPV.*
