# Supplementary figures and images for: Needle-free delivery of measles virus vaccine to the lower respiratory tract of non-human primates elicits optimal immunity and protection
Source: NPJ Vaccines. 2017 Aug 1;2:22. doi: 10.1038/s41541-017-0022-8 (PMC5627256; doi:10.1038/s41541-017-0022-8)

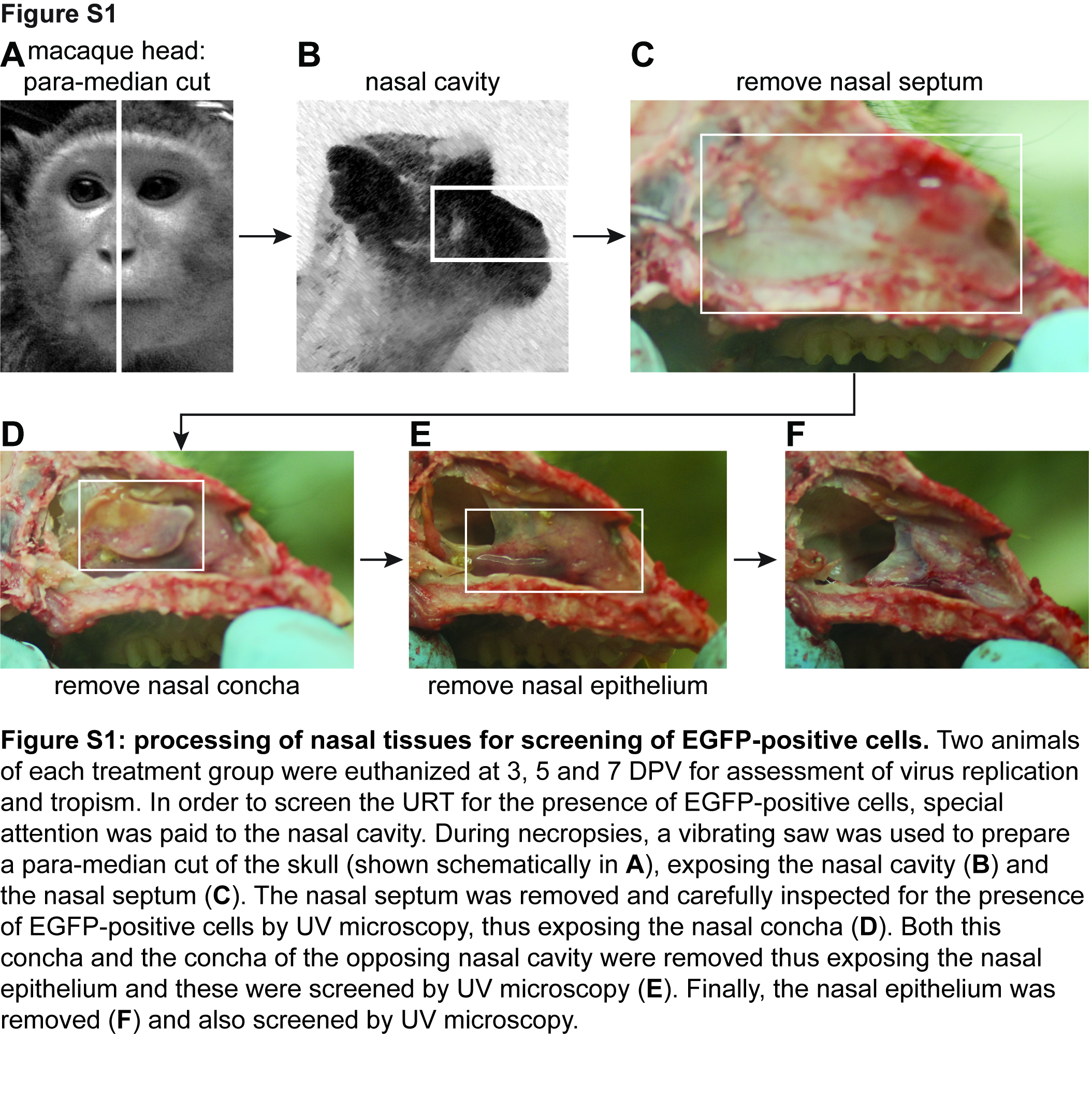

Supplement: Supplementary file 2 — Figure S1 [file 41541_2017_22_MOESM2_ESM.tif]

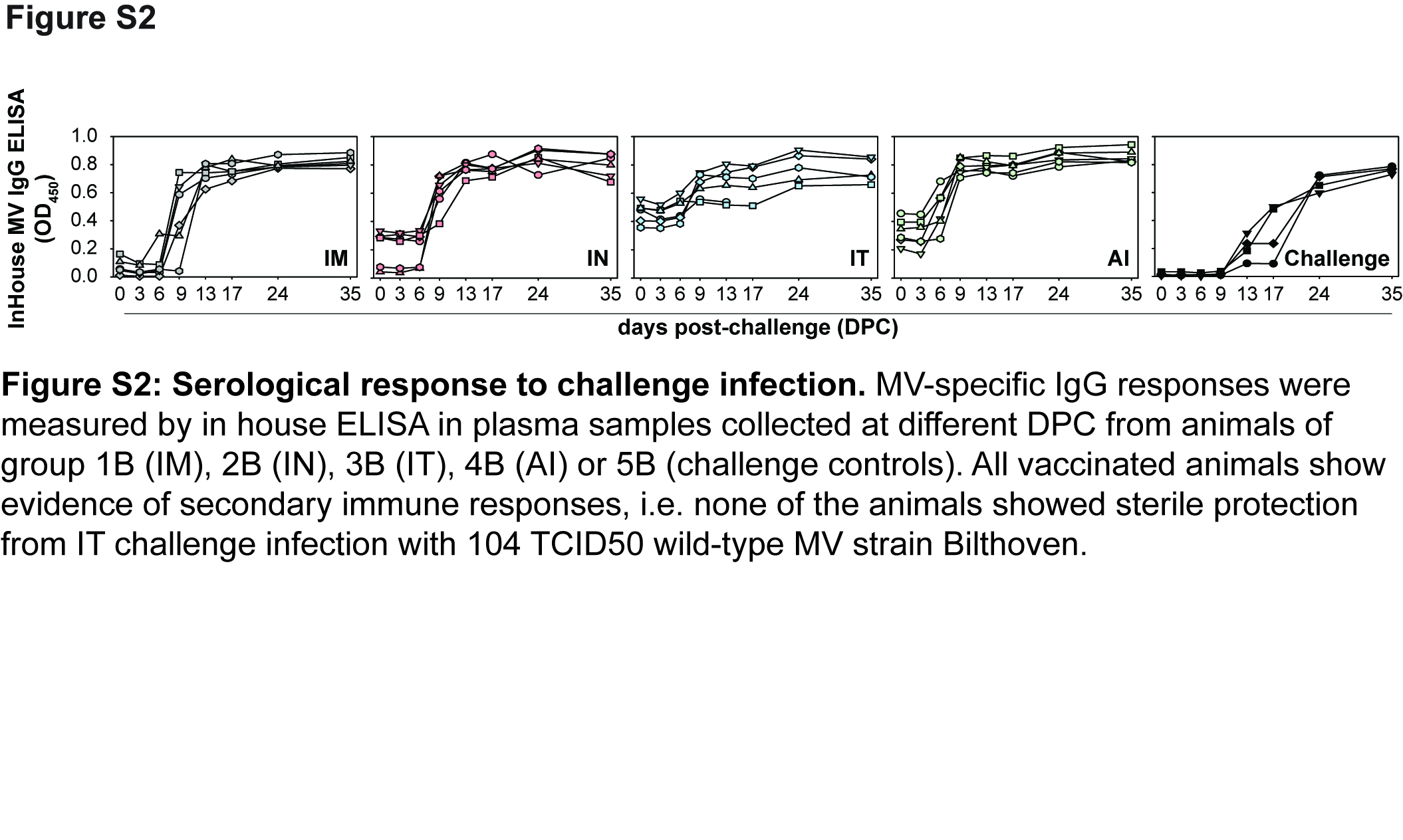

Supplement: Supplementary file 3 — Figure S2 [file 41541_2017_22_MOESM3_ESM.tif]
